# Optimized Protocols to Extract Total Transcripts and Proteins from Lipid-Rich Tissues

**DOI:** 10.3390/mps9020065

**Published:** 2026-04-10

**Authors:** Nicolas De Azevedo, Anthony Lozano, Ramon E. Parsons, Tiphaine C. Martin

**Affiliations:** 1 Tisch Cancer Institute, Department of Oncological Sciences, Icahn School of Medicine at Mount Sinai, New York, NY 10029, USA; nicolas.deazevedo@mssm.edu (N.D.A.); anthony.lozano@mssm.edu (A.L.); ramon.parsons@mssm.edu (R.E.P.); 2 Department of Immunology and Immunotherapy, Icahn School of Medicine at Mount Sinai, New York, NY 10029, USA; 3 Division of Liver Diseases, The Icahn School of Medicine at Mount Sinai, New York, NY 10029, USA; 4 Department of Genetics and Genomic Sciences, Icahn School of Medicine at Mount Sinai, New York, NY 10029, USA; 5 Center for Engineering and Precision Medicine, Icahn School of Medicine at Mount Sinai, New York, NY 10029, USA

**Keywords:** lipid-rich tissues, adipose, brain, liver, protein, transcript, RT-qPCR, RNA-seq, RNA

## Abstract

Background: Highly lipidic tissues (e.g., adipose tissue, brain, and liver) are challenging for transcript and protein extraction and for next-generation sequencing. Lipids can clog filters, columns, and pipettes; cause autofluorescence and quenching in imaging; and interfere with centrifugation-based separation. Aim: To identify the most suitable method for extracting total RNA for RT-qPCR and an alternative method for extracting total protein for quantification in mice fed a regular or high-fat diet. Methods: We compared three total RNA extraction methods and two total protein extraction methods. Results: The highest total RNA yield and purity were obtained with TRIzol and chloroform, with optimized steps added to the original protocol to address the challenges posed by highly lipid-rich tissues. For total protein extraction, an adipose tissue-specific kit from Invent Biotechnologies yielded higher protein levels than the classical RIPA-based method. Among lipid-rich tissues, we observed that adipose tissue was more challenging to process than the brain and the liver. Conclusions: Adipose tissue, particularly under a high-fat diet, is the most challenging lipid-rich tissue, followed by the brain and then the liver. We highlight protocols that improve total RNA and protein yields and purity, which may benefit other researchers working with these tissues.

## 1. Introduction

Obesity has become a major global health issue, affecting millions of people worldwide. Obesity is a medical condition characterized by excessive body fat that increases the risk of serious health problems. Obesity is linked to conditions such as heart disease, type 2 diabetes, and high blood pressure and is influenced by factors like poor diet, lack of physical activity, and genetics. In many countries, obesity-related illnesses and cardiovascular disease are among the leading preventable causes of death. Still, their prevalence has tripled since 1975 in both developed and developing countries, among adults and children. It significantly impacts the economy, both directly through healthcare costs (such as hospitalization, medications, and surgeries) and indirectly through lost productivity, disability, and premature death. GLP-1 and GLP-1R agonist drugs, which mimic natural gut–brain signaling rather than artificially stimulating metabolism or suppressing appetite, reduce body weight by up to 20% over 1 year and reduce major cardiovascular events by 20% without changing patient habits [1,2]. However, a drawback of GLP-1 agonists is the reduction in lean and muscle mass, which is often not recovered after discontinuation [3]. Moreover, when patients discontinue treatment, as with other approaches that focus on reducing body weight, they quickly regain weight and the associated risks, highlighting the need to study adipose, brain, and liver tissue to identify new mechanisms and targets for therapies that can complement these treatments, such as the immune system [4,5].

The study of multi-omics markers in tissues is essential for diagnosing diseases, developing prognostic and predictive models, and understanding the altered mechanisms underlying disease, potentially leading to new therapies. Initially, biomarkers are created and identified in blood because blood collection is less invasive and sample management is well established. To verify that these blood biomarkers accurately reflect the disease and affected tissues, and to explore their mechanisms, researchers must examine the relevant tissues. Different types and locations of white adipose tissue have been identified, each with unique links to diseases; therefore, they have distinct functions [6,7,8,9]. In addition, several other tissues are rich in lipids (Table 1), with similar lipid percentages observed in both mice and humans. White adipose tissue is the most lipid-rich tissue, and its lipid content increases with a high-fat diet.

Although extensive studies on obesity and cardiovascular disease have been conducted, adipose tissue remains relatively understudied in humans, and no clear protocols have been established to overcome the challenges described below. Several factors complicate the study of highly-lipidic tissues. Very lipid-rich tissues have lower transcript and protein levels per cell size than low-lipid tissues [23]. Another challenge is lipid content. Indeed, lipids cause autofluorescence and quenching in imaging [24]; clog filters, columns, and pipette tips; and interfere with enzymatic reactions [25,26]. In tissues with high lipid content, buoyant adipocytes that float during spinning can hinder separation and cause clogging [27]. Cells with high intracellular lipid content are more prone to rupture, leading to mechanical fragility and lower single-cell yields [28]. Lipids in cells can contaminate phases and inhibit reverse transcription and enzymatic reactions, reducing the quality and quantity of DNA, RNA, and proteins [29,30,31].

We developed and tested protocols for extracting total RNA and proteins from adipose tissue after either a normal or a high-fat diet. We tested three commercial methods for extracting total transcripts and two methods for protein extraction from white adipose tissue and from two other lipid-rich tissues (liver and brain) under both diets to identify the most appropriate methods and assess whether challenges differ across tissues. These comparisons showed that white adipose tissue is the most challenging tissue under a high-fat diet, and two murine knockout models were more challenging. For RNA extraction, the three commercial methods yielded unsatisfactory metrics, but the TRIzol protocol allowed for tuning. We proposed an optimized TRIzol protocol for isolating total RNA from highly lipid-rich tissues. We identified a specific protein extraction method for adipose tissue from Invent Biotechnologies that is most suitable for analyzing highly lipid-rich tissues. Although some differences between mice and humans have been noted in adipose tissue [32], the mouse remains a standard preclinical model for studying human diseases, including white adipose tissue, before clinical trials. The protocols were developed using mouse tissues but could also be applied to human tissues.

## 2. Experimental Design

**Figure 1:**
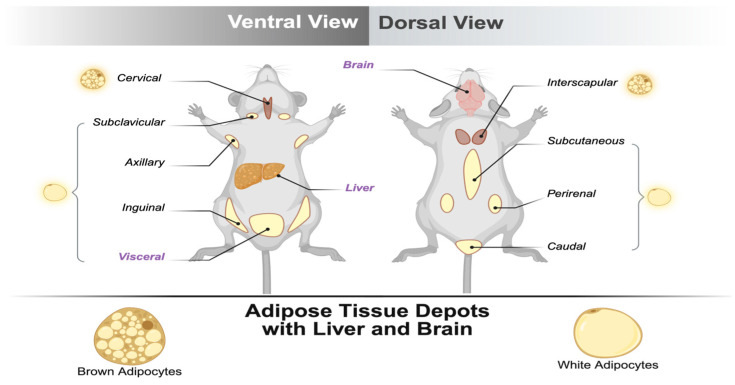
Highly lipid-rich tissues. The tissues written in purple were tested in this protocol.

The general experimental design and the time required to complete each stage are presented in Figure 1. We tested three protocols for total RNA extraction and two for total protein extraction. Although all five protocols performed well for the liver and the brain, we found that only the optimized protocol combining TRIzol and chloroform to collect transcripts and the kit specifically for adipose tissues from Invent Biotechnologies to collect proteins performed well for adipose tissues from HFD. If you observe low yield from your adipose tissues, we recommend applying the same protocol to the liver, which is an easier tissue to obtain good concentration and quality with these protocols, to determine whether the issue lies with your reagents or with other factors.

### 2.1. Materials

1. TRIzol^TM^ Reagent (Invitrogen, Waltham, MA, USA; Cat. no.: 15596026)
2. RNeasy Plus Mini Kit (Qiagen, Redwood City, CA, USA; Cat. no.: 74134)
3. RNeasy Lipid Tissue Mini Kit (Qiagen, Redwood City, USA; Cat. no.: 74804)
4. Minute Total Protein Extraction Kit for Adipose Tissues/Cultured Adipocytes (Invent Biotechnologies, Plymouth, MN, USA, Cat. No.: AT-022)
5. Chloroform (Fisher Chemical, Emeryville, CA, USA; Cat. no.: C606-4)
6. Isopropanol (Thermo Scientific Chemical, Emeryville, CA, USA; Cat. no.: 423830040)
7. Ethanol (Greenfield Global, Brookfield, WI, USA; Cat. no.: P111WORLD200CS4L)
8. RNase-free water (Qiagen, Redwood City, USA; Cat. no.: 129112)
9. Allprotect Tissue Reagent (Qiagen, Redwood City, USA; Cat. no.: 76405)
10. RIPA buffer (Sigma Aldrich, Burlington, VT, USA; Cat. no.: R0279-500 mL)
11. Halt^TM^ Protease and Phosphatase Inhibitor Cocktail (100×) (Thermo Fisher Scientific, Emeryville, CA, USA; Cat. no.: 78440)
12. Microcentrifuge Tube 5 mL (Thomas Scientific, Swedesboro, NJ, USA; Cat. no.: 1191K69)
13. Low-Protein Binding Microcentrifuge Tube 1.5 mL (Eppendorf, Edison, NJ, USA; Cat. no.: EPPR4513)
14. Low-Protein Binding Microcentrifuge Tube 2 mL (Posiclick Thomas Scientific, Swedesboro, NJ, USA; Cat. no.: 1149Y01)
15. UltraPure Distilled water DNase-free and RNase-free (Invitrogen, Waltham, USA; Cat. no.: 10977-015)
16. Ethanol, Molecular Biology Grade (200 Proof) (Fisher BioReagents, Pittsburgh, PA, USA, Cat. No.: BP2818500)
17. Qubit RNA BR Assay Kit (Thermo Fisher Scientific, Emeryville, USA; Cat. No.: Q10210)
18. Optical tube strip caps (8× strip) (Agilent, Santa Clara, CA, USA; Cat. No: 401425)
19. Optical tube strips (8× Strip) (Agilent, Santa Clara, USA; Cat. No: 401428)
20. Loading Tips, 1 Pk (Agilent, Santa Clara, USA; Cat. No: 5067-5598)
21. High-Sensitivity RNA ScreenTape (Agilent, Santa Clara, USA; Cat. No: 5067-5579)
22. High-Sensitivity RNA ScreenTape Ladder (Agilent, Santa Clara, USA; Cat. No: 5067-5581)
23. High-Sensitivity RNA ScreenTape Sample Buffer (Agilent, Santa Clara, USA; Cat. No: 5067-5580)

### 2.2. Equipment

- Bullet Blender^®^ 5E Pro (Next Advance, Seattle, WA, USA; Cat. no.: BT5E)

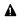

**NOTE:** The Bullet Blender^®^ Homogenizer is designed to work only with an Eppendorf 5 mL microtube.

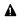

**NOTE:** An alternative, lower-cost option is the TissueRuptor II (Qiagen, Redwood City, USA, Cat. No.: 9002755). This method allows processing of only one sample at a time; the sample must be kept cold on ice or in a refrigerator to prevent RNA degradation. We observe a slightly lower concentration than with the Bullet Blender Homogenizer. You need at least 3 mL of buffer to disrupt the tissues.

- NanoDrop 2000 spectrophotometer (Thermo Fisher Scientific, Emeryville, USA; Cat. no.: ND-2000)
- Tungsten Carbide beads 3 mm (Qiagen, Redwood City, USA; Cat. no.: 6997)
- Microcentrifuge that could be at 4 °C, such as SorVall Legend Micro 17R Centrifuge (Thermo Fisher Scientific, Emeryville, USA; Cat. no.: 75002431)
- Tweezers
- Scalpel
- Agilent 4150 TapeStation (Agilent, Santa Clara, USA; Cat. No: G2992AA)
- Qubit 4 fluorometer (Thermo Fisher Scientific, Emeryville, USA; Cat. No.: Q33238)

## 3. Procedure

### 3.1. Animals

Before collecting tissues from animals, mice may be on a regular diet or a high-fat diet (HFD). The study involved 18-week-old male C57Bl/6 mice, some of which had been on an HFD for 10 weeks, from three genotypes (wild-type, Cd68^−/−^ [33], and Ptenl^−/−^ [34]); they were used to test protocols under highly lipidic conditions. The mice had to be euthanized in accordance with an approved protocol developed by the Institutional Animal Care and Use Committee (IACUC). In this study, we used 5 min CO_2_ exposure at a flow rate of 5 L/min to induce rapid unconsciousness, followed by cervical dislocation. All animals were housed, fed, and treated in accordance with the IACUC guidelines and regulations at the Icahn School of Medicine at Mount Sinai. 

### 3.2. Organ Collection

This study highlighted protocols for extracting transcripts and proteins from epididymal visceral adipose tissue, liver, and brain (Figure 1). The protocols for organ collection are briefly detailed here; several previous papers provide more detail. We list a few here, which are detailed protocols for collecting adipose tissue from adults [35] and mouse pups [36], liver tissue [37], and brain tissue from adults [38,39] and pups [40].

Following euthanasia (5 min CO_2_ followed by cervical dislocation), the abdominal cavity was opened using a T shape under sterile conditions. First, the epididymal white adipose depot was carefully excised, cleared of visible connective tissue and lymph nodes, briefly rinsed in ice-cold phosphate-buffered saline (PBS), weighed, and snap-frozen in liquid nitrogen or in dry ice with 100% ethanol for at least 3 min for later downstream analyses. Liver was then rapidly excised, rinsed in ice-cold PBS to remove excess blood, sectioned as required, and snap-frozen in liquid nitrogen or in dry ice with 100% ethanol for at least 3 min. For brain collection, the skull was carefully opened using fine scissors, and the brain was gently removed. Whole brains were snap-frozen in liquid nitrogen or in dry ice with 100% ethanol for at least 3 min. All tissues were stored at −80 °C until further processing unless otherwise specified.

### 3.3. Protocols to Extract Total Transcripts

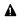

**CRITICAL STEP:** The following steps must be performed under a hood to reduce exposure to toxic components of chloroform and phenol.

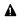

**CRITICAL STEP:** If you begin with frozen tissue, keep it frozen by placing it in a liquid at −120 °C (liquid nitrogen or 100% ethanol on dry ice) to minimize tissue degradation and preserve its overall structure. If you are working with fresh tissue, keep it at 4 °C to slow tissue degradation.

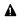

**CRITICAL STEP:** You can place a portion of your samples from tissues other than adipose tissue in RNAlater RNA stabilization reagent at room temperature (21–23 °C). A better alternative is Allprotect Tissue Reagent, which provides immediate stabilization of DNA, RNA, and proteins in tissue samples at room temperature (21–23 °C) and is best stored at −20 °C for 2–3 months storage or −80 °C for 1 year storage, as low temperatures inhibit RNases and prevent RNA and protein degradation.

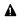

**CRITICAL STEP:** All materials must be kept cold (e.g., at 4 °C using ice), and the work must be performed quickly to prevent RNA degradation under aseptic, RNase-free conditions.

#### 3.3.1. Before Starting Any Protocols: 30 min

1. Tidy up the work area.
2. Aseptically clean the space using RNase Zip and 70% ethanol in RNase-free water to eliminate ribonucleases (RNases), which degrade RNA, and to inactivate microbes by denaturing proteins.
3. Use new boxes of tips and wipers.
4. Prepare tubes with labels, Petri plates, and razor blades.
5. Clean racks, pipettes, pipets, forceps, a centrifuge, and a Bullet Blender^®^ Homogenizer with RNase Zip and 70% ethanol in RNase-free water to eliminate RNases and inactivate microbes by denaturing proteins.
6. Put TRIzol and tubes on ice to maintain reagent homogeneity and stability.

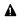

**NOTE:** Freezing TRIzol may alter components, such as phenol, causing crystallization or precipitation and reducing extraction efficiency.

7. Set the centrifuge to 4 °C to minimize degradation by inhibiting RNase activity and limiting chemical degradation during phase separation, as high-speed centrifugation generates heat.
8. Keep the Bullet Blender^®^ Homogenizer at 4 °C when in use to minimize degradation by inhibiting RNase activity and limiting chemical degradation during phase separation, as high-speed centrifugation generates heat.
9. Prepare dry ice to keep the tissue frozen, or ice on which to keep the fresh tissue.

#### 3.3.2. Optimized Protocol Combining TRIzol and Chloroform: 2 h Plus Time to Collect Tissues (Max 10 min)

**Figure 2:**
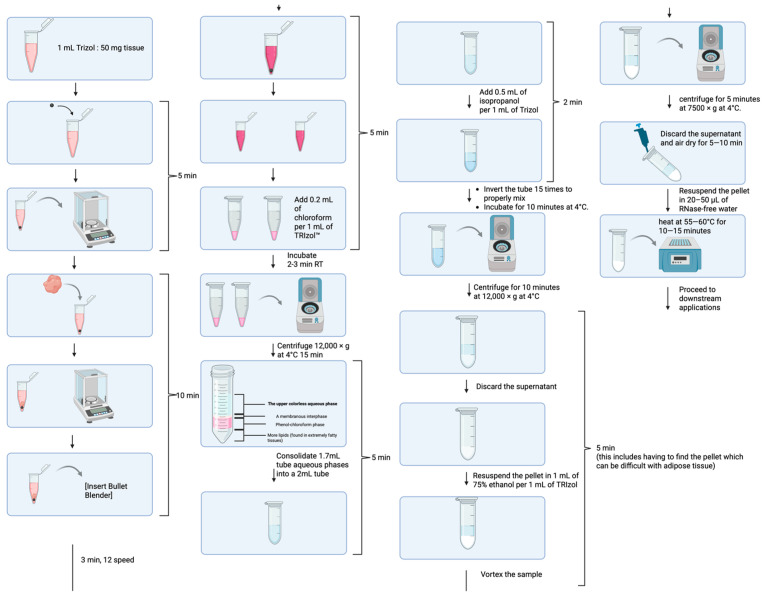
Steps of our optimized protocol combining TRIzol and chloroform. Arrows indicate the order of steps.

The main steps of our optimized TRIzol-based protocol, which contains phenol and guanidinium isothiocyanate and chloroform, are presented in Figure 2 and described below.

##### Separation of Tissues into Phases Based on Their Compositions (26–38 min)

10. Add 1 mL of TRIzol reagent per 50 mg of tissue in a 5 mL tube. Trizol contains phenol, which facilitates lysis of fat cells, denatures proteins, and protects RNA from RNase degradation by inhibiting RNase activity.

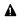

**CRITICAL STEP:** For measuring tissue weight, especially adipose tissue, calculate the difference between the 5 mL tube containing already 1 mL of TRIzol with beads and the tube containing the sample. Because adipose tissue is highly lipidic tissue, it liquefies rapidly, which can lead to loss of valuable RNA during transport from a weigh boat to a tube.

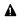

**CRITICAL STEP**: We determined that the minimum initial tissue amount for a satisfactory yield is 80 mg.

11. Add one or two tungsten beads to the TRIzol in 5 mL tubes.
12. Input 5 mL tubes containing samples in the Bullet Blender^®^ Homogenizer.

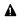

**NOTE:** The placement and number of samples do not need to be symmetrical in the Bullet Blender^®^ Homogenizer, due to the homogenizer not employing a centrifuging action.

13. Run at speed 12 for 3 min if you are using Bullet Blender^®^ 5E Pro (Next Advance USA; Cat. no.: BT5E).

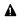

**NOTE**: Do not stay in the cold room while the machine is running, as there is a risk of ear damage from the noise. Inform others not to enter the cold room.

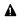

**NOTE**: If you have another Bullet Blender^®^, speed 12 is the highest speed for the Bullet Blender^®^ 5E Pro (Next Advance USA; Cat. no.: BT5E).

**Figure 3:**
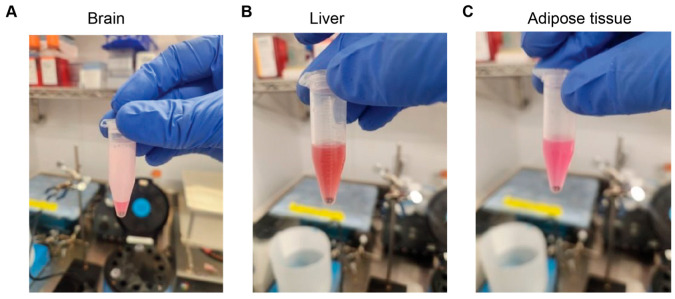
Lysate of Different Lipidic Tissues Utilizing Bullet Blender^®^ Homogenizer. From left to right: (**A**) brain, which produces more foam than other tissues; (**B**) liver, which has more red blood cells, and the solution is redder as a consequence; (**C**) adipose.

14. If tissue remains visible in the TRIzol (Figure 3), run the sample in the Bullet Blender^®^ Homogenizer again for 2 min, as this indicates incomplete tissue degradation. Repeat for 2 min, running until the tissue is fully degraded.

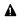

**NOTE**: In the case of brain tissue, be aware that foaming is highly prominent, and it takes more time for the foam to become liquid.

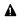

**NOTE**: In the case of liver tissue, be aware that the solution may appear more reddish because of blood.

15. Incubate at room temperature (21–23 °C) for 5 min to allow complete dissociation of the nucleoprotein complex by the TRIzol.
16. **OPTIONAL STEP:** If samples have a high fat content or are adipose tissues, centrifuge the lysate for 5 min at 16,000× *g* at 4 °C to more effectively separate the lysate from tissue fragments, then transfer the clear supernatant to a new tube.
17. **OPTIONAL STEP:** If step 16 is performed, centrifuge again at 1200× *g* for 5 min at 4 °C.
18. **OPTIONAL STEP:** If step 16 is performed, remove the supernatant layer containing the lipid.

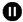
 **PAUSE STEP:** Disrupted tissue in TRIzol solution can be stored at –20 °C for 2–3 months, −80 °C for up to 1 year, or at 4 °C overnight.

19. Transfer 1–2 mL of TRIzol-treated disrupted tissue into two 1.7 mL tubes.
20. Add 0.2 mL of chloroform per 1 mL of TRIzol^TM^ Reagent to extract lipids from aqueous samples.

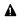

**NOTE:** Chloroform is an organic solvent that is denser than water and is immiscible with water-based samples. It facilitates phase separation by interacting with TRIzol at acidic pH during centrifugation, thereby separating RNA from DNA and proteins.

21. Securely cap the tube, then thoroughly mix by shaking for 15 s.
22. Incubate at room temperature (21–23 °C) for 2–3 min.

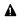

**CRITICAL STEP:** These two steps enable full interaction between chloroform and TRIzol, thereby improving phase separation.

**Figure 4:**
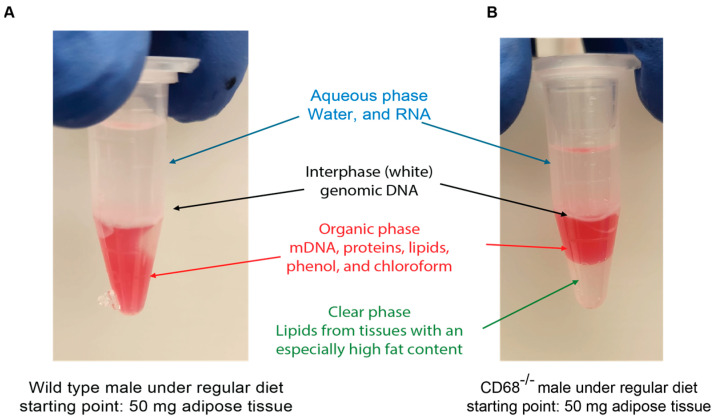
Phases produced by the phenol–chloroform complex. (**A**) Three phases were observed in wild-type mice on a regular diet. (**B**) Four phases were observed in CD68^−/−^ mice on a regular diet.

**Figure 5:**
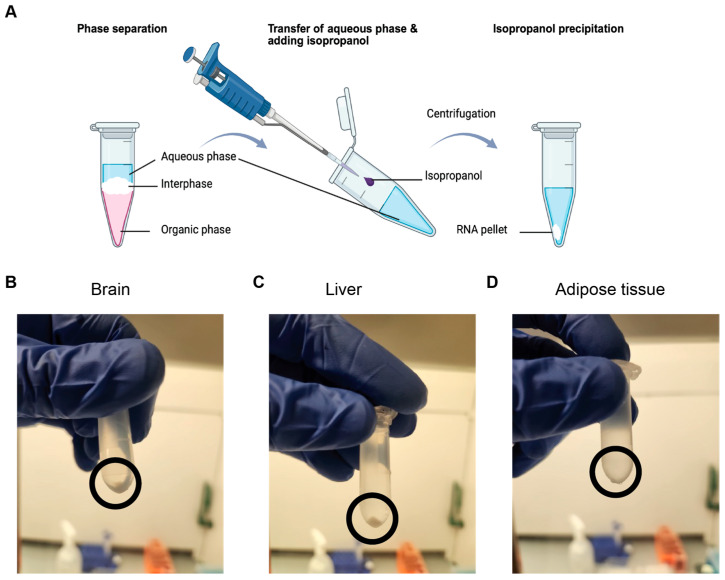
Pellet of total RNA. (**A**) Schematic showing the steps from phase separation to isopropanol-based RNA precipitation, used to isolate RNA pelleted from the aqueous phase. Here, arrows indicate the transfer to the next step. The last transfer is also followed by centrifugation step to obtain the RNA pellet. (**B**–**D**) Representative images of RNA pellets after isopropanol precipitation and centrifugation. Black circles highlight the pellets for the (**B**) brain; (**C**) liver (with the highest concentration and clearly visible); and (**D**) adipose tissue (with the lowest concentration).

23. Centrifuge the sample at 16,000× *g* for 15 min at 4 °C to separate the mixture into phases.
24. Once completed, there should be 3–4 phases present (Figure 4): (1) the upper colorless aqueous phase, which contains water and RNA; (2) a membranous interphase, which contains more genomic DNA; (3) a lower red phenol–chloroform phase, which contains mitochondrial DNA, proteins, lipids, phenol, and chloroform; and (4) a transparent layer that can appear in extremely fatty tissues.
25. Transfer the RNA-containing aqueous phase from each tube for the same tissue sample into a single 2 mL tube by tilting the tube at 45° and carefully pipetting the solution (Figure 5A).

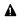

**NOTE**: It is essential to avoid collecting any interphase or organic layer.

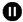
 **PAUSE STEP:** The RNA-containing aqueous phase can be stored at –20 °C for 2–3 months or −80 °C for up to 1 year.

##### Isolating RNA (30 min)

26. Precool the isopropanol by keeping it at 4 °C before use for at least 30 min.
27. Add 0.5 mL of precooled isopropanol per 1 mL of TRIzol^TM^ reagent to the aqueous phase and keep the tube at 4 °C.

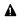

**NOTE**: Precooled isopropanol can maintain the aqueous phase at a low temperature, preventing RNA degradation.

28. Invert the tube 15 times to properly mix, enabling full interaction between the isopropanol and RNA.
29. Incubate for at least 15 min (up to overnight) at 4 °C or even −20 °C, but no more than 15 min.

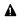

**CRITICAL STEP**: Cold temperatures (4 °C or −20 °C) decrease the solubility of nucleic acids in the alcohol–salt mixture, thereby enhancing precipitation efficiency and increasing yield, particularly for low-concentration samples. While using 4 °C or −20 °C boosts yield, it also raises the risk of salt co-precipitation, making a thorough wash with 75% ethanol essential.

30. Centrifuge for 15 min at 12,000× *g* at 4 °C to precipitate RNA.

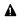

**NOTE**: Guanidinium salt binds to the RNA backbone with the help of isopropanol. Total RNA precipitates as a white, gel-like pellet at the bottom of the tube. In liver tissue, a pellet will be visible at the bottom after centrifugation. However, in adipose and brain tissue, a pellet is rarely seen at this step, and the former is even less likely to produce a proper visible pellet.

31. Circle with marker pellet/possible spot for pellets (see Figure 5).
32. Discard the supernatant using a micropipette, ensuring that the pellet is not pipetted.

##### Washing the RNA (1 h)

33. Resuspend the pellet in 1 mL of 75% ethanol per 1 mL of TRIzol^TM^ reagent used for lysis.

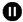
 **PAUSE STEP**: RNA can be stored in 75% ethanol at −20 °C for 2–3 months or −80 °C for up to 1 year.

34. Vortex the insoluble pellet for about 30 s to facilitate desalting wash.
35. Centrifuge for 5 min at 12,000× *g* at 4 °C.

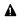

**NOTE**: A higher centrifugal speed of 12,000 × g instead of 7500 × g precipitates the RNA more efficiently, and this is important for adipose tissues, as there is less RNA per cell. At this stage, only brain and liver tissue have been shown to consistently produce pellets.

36. Discard the supernatant with a micropipette, then use a 20 µL tip to remove most of the ethanol.

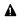

**CRITICAL STEP**: For adipose tissue or a low-purity sample, repeat the wash steps 33–36 2–3 times to remove impurities more thoroughly.

37. Vacuum or air-dry the RNA pellet for 5–10 min. As it dries, if the pellet is effectively desalinated, it will become transparent.
38. Resuspend the pellet in 20–50 µL of RNase-free water.
39. Incubate in a water bath or heat block set at 55–60 °C for 10–15 min.

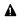

**CRITICAL STEP**: The heating step allows the remaining ethanol to evaporate.

40. Pipette up and down again, then leave on ice for a further 30 min to ensure the RNA completely dissolves.
41. Measure the concentration and purity of samples.

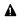

**NOTE**: One way to ensure a cleaner sample is to reprecipitate it, then wash with ethanol, extend air-drying, and resuspend in a fresh volume of TE or pure RNA-free water. The reported concentration may be significantly lower than before because you have successfully removed contamination that affected your absorbance data.

42. Proceed to downstream applications, or store the RNA at −20 °C for 2–3 months or –80 °C up to 1 year.

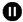
 **PAUSE STEP**: RNA dissolved in water can be stored at −20 °C for 2–3 months or −80 °C for up to 1 year.

#### 3.3.3. RNeasy Lipid Tissue Mini Kit: 45 min with Time to Collect Tissues (Max 10 min)

We followed the Qiagen protocol for the RNeasy Lipid Tissue Mini Kit [41], optimized for the lysis of tissues rich in fat and the purification of high-quality total RNA with nucleotide sizes greater than 200. Still, it can also be used for other tissues. The procedure enriches for RNA because most RNAs with nucleotide lengths below 200 (such as 5.8S rRNA, 5S rRNA, and tRNAs, which together comprise 15–20% of total RNA) are selectively excluded. For the purification of small RNAs, including microRNAs, from tissues and cells, they recommend the use of miRNeasy Kits. The RNeasy Lipid Tissue Mini Kit integrates a phenol- and guanidine-based lysis buffer, silica-membrane-based total RNA purification in centrifuge tubes, and chloroform for RNA extraction. We present only the critical steps we identified as important after evaluating their protocol but which were missing or unclear.

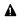

**CRITICAL STEP:** Do not exceed 100 mg of starting tissue; exceeding this amount will not increase RNA yields and may even decrease them. This phenomenon happens because the binding capacity of the RNeasy Mini spin column is surpassed.

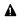

**CRITICAL STEP:** Ensure the starting material is completely lysed before proceeding to the next step, as incomplete lysis may reduce RNA yields.

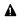

**CRITICAL STEP:** Ensure that the dissolved RNA from the last step is transferred into a 1.5 mL tube that can be properly closed to minimize water evaporation.

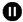
 **PAUSE STEP**: RNA dissolved in water can be stored at −20 °C for 2–3 months or at −80 °C for up to 1 year.

#### 3.3.4. RNeasy Plus Mini Kit: 45 min with Time to Collect Tissues (Max 10 min)

We followed the Qiagen RNeasy Plus Mini Kit protocol [42], which is the generic Qiagen protocol for purifying total RNA from animal cells, animal tissues, bacteria, and yeast, as well as for RNA clean-up from small amounts of starting material.

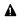

**CRITICAL STEP**: Do not use more than 30 mg of starting tissue; exceeding this amount will not increase RNA yields and may even decrease them. If a larger starting point is needed, there are other kits from the same company (RNeasy Plus Midi Kit and RNeasy Plus Max Kit).

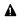

**CRITICAL STEP**: Ensure that the dissolved RNA from the last step is transferred into a 1.5 mL tube that can be properly closed to minimize water evaporation.

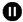
 **PAUSE STEP**: RNA dissolved in water can be stored at −20 °C for 2–3 months or at −80 °C for up to 1 year.

#### 3.3.5. After Extracting Total RNA

1. Measure the concentration of total RNA using Nanodrop and check the UV light absorbance at 230 nm (pure nucleic acids absorb UV light), 260 nm (pure nucleic acids absorb UV light), and 280 nm (pure proteins absorb UV light), which are used to measure the concentration and purity of DNA and RNA.

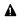

**NOTE**: The ratio of absorbance at 260 nm to 280 nm (A_260/280_) is a measure of protein contamination (e.g., below 2 for RNA). The ratio of absorbance at 260 nm to 230 nm (A_260/230_) indicates the presence of organic compounds or salts, like phenol, guanidine salts, or EDTA (e.g., below 2 for RNA). For pure RNA, the A_260/280_ ratio should be around 2.0, and the A_260/230_ ratio should be approximately 2.0–2.2. Some methods, such as microarrays and RNA-seq, are sensitive to A_260/230_ ratios because carryover chemicals from the extraction can increase background. However, RT-qPCR is not sensitive to low A_260/230_ ratios; however, a ratio above 1 is still helpful.

2. If you do not have a Nanodrop, you can also use a Qubit to measure RNA concentration. Choose the appropriate kit based on the potential concentration of your samples.
3. Check the integrity of the extracted RNA by agarose gel electrophoresis [43] or an Agilent TapeStation.

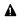

**CRITICAL STEP:** This step is essential for downstream RNA-seq or RT-qPCR analysis. Please perform it within a reasonable timeframe to get good RNA integrity.

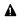

**NOTE:** After a few months at −20 °C and longer at −80 °C, you can observe a drop in the concentration and RNA integrity. Please double-check the concentration and RNA integrity of your RNA pellets just before downstream RNA-seq or RT-qPCR analysis.

### 3.4. Protocols to Extract Total Proteins

#### 3.4.1. Before Starting Any Protocols: 30 min

1. Clean the work area.
2. Prepare tubes with labels, Petri plates, and razor blades.
3. Put tubes on ice to maintain sample integrity.
4. Set the centrifuge at 4 °C to prevent protein degradation, minimize enzymatic activity, and manage heat generated during high-speed rotation.
5. Prepare dry ice to keep the tissue frozen, or use ice to keep the tissue fresh.

#### 3.4.2. Extraction of Proteins with Minute Total Protein Extraction Kit for Adipose Tissues/Cultured Adipocytes

We followed the protocol of Invent Technology’s “Minute Total Protein Extraction Kit for Adipose Tissues/Cultured Adipocytes”, as detailed below.

1. Pre-chill buffer A and the filter cartridge in the collection tube on ice to maintain buffer integrity and prevent protein degradation during sample contact.
2. Add protease and phosphatase inhibitors to buffer A to prevent protein degradation and phosphorylation site modification.
3. Follow steps 2–6 from the protocol of Invent Technology’s “Minute Total Protein Extraction Kit for Adipose Tissues/Cultured Adipocytes”, section “Protein Extraction from Adipose Tissues (WAT or BAT)”.
4. The sample can also be resuspended in buffer B or C to dissolve the water-insoluble proteins for downstream applications.
  4.1. Add 1/10 of Buffer B to the extracted protein solution, yielding a denatured protein solution for Western blotting as we did here.
  4.2. Add 1/10 of Buffer C to the extracted protein solution to obtain a non-denatured protein solution for the Luminex or ELISA assay, as we did in our research paper. We could perform the Luminex or ELISA assay without adding Buffer C.

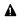

**NOTE:** We obtained additional information on their buffers from the technical team at Invent Technology, which may assist you in selecting the most suitable buffer for your experiment.

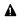

**NOTE**: Buffer A is a proprietary, detergent- and EDTA-free buffer that gently lyses cells, releases soluble cytoplasmic proteins, and preserves proteins in their native state, including their 3D structure and protein–protein interactions, while maintaining their functional activity. This buffer helps prevent interference in downstream applications sensitive to these reagents. All chemicals in Buffer A have molecular weights below 100 Daltons.

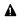

**NOTE**: Buffer B contains 2.5% SDS, which lyses cell membranes, denatures proteins, and solubilizes them, especially membrane-bound or aggregated proteins. It disrupts tertiary and secondary structures, coating proteins with a negative charge to enable separation by molecular weight during electrophoresis.

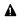

**NOTE**: Buffer C contains 5% NP-40 and Triton X-100, which efficiently solubilize membrane-bound proteins, lyse cytoplasmic membranes, and release soluble proteins while preserving protein–protein interactions and native structures.

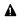

**NOTE:** To troubleshoot protein extraction and purification, we advise consulting the protocol provided by Invent Technology Company.

#### 3.4.3. Extraction of Proteins with RIPA Buffer: 15 min per Sample

5. Add protease and phosphatase inhibitors to the RIPA buffer to prevent protein degradation and phosphorylation site modifications.
6. Keep the modified RIPA buffer on ice.
7. Prepare the fresh tissue by rinsing it in chilled PBS and cutting it into small pieces. You can also do this with frozen tissues. Keep the tissues on ice.
8. Combine the minced tissue with pre-chilled RIPA lysis buffer at approximately 10 mL of buffer per 1 g of tissue. Use a smaller volume if a more concentrated extract is needed. So, 1 mL of RIPA buffer for 100 mg of tissue.
9. Stir the mixture at high speed for several minutes until no tissue fragments remain, ensuring homogeneity.

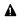

**CRITICAL STEP:** We observed a limitation with either the Bullet Blender^®^ Homogenizer or the tissue disrupter: it requires at least 3 mL of buffer to operate, which corresponds to 300 mg of tissue. This is not possible in our murine adipose tissue model.

10. Place the tube on ice for about 30 min to allow complete tissue lysis.
11. Centrifuge the tube at high speed (e.g., 10,000× *g*) for 10 min at 4 °C.
12. Carefully transfer the clear supernatant containing the extracted proteins to a new tube for downstream applications.

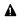

**NOTE**: We did not pursue protein extraction using RIPA buffer because we did not have 300 mg of tissue for all samples, which would limit further large-scale studies.

#### 3.4.4. After Extracting Proteins

13. Measure protein concentration using a BCA assay.

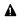

**NOTE**: The Bradford assay is not compatible with the Minute kit.

## 4. Expected Results

### 4.1. RNeasy Plus Mini Kit

We tested the kit on WT mice after an HFD. We observed an increase in concentration with increasing tissue number (Table 2). However, there is no linear relationship. Interestingly, the kit states that the tissue limit is 30 mg. We found that exceeding this limit yields a slightly higher final total RNA concentration, but the total RNA yield per tissue sample is reduced. With sufficiently pure total RNA, the concentration we obtained is suitable for RNA sequencing, as it can be reduced to the picogram range. However, it is insufficient for RT-qPCR with the SYBR Green kit on 96-well plates, which requires at least 91 ng/μL.

### 4.2. RNeasy Lipid Tissue Mini Kit

We decided to test the RNeasy Lipid Tissue Mini Kit, which is claimed to be specific for highly lipidic tissues. First, we applied it to WT mice after an HFD (Table 3). We observed higher concentrations than with the RNeasy Plus Mini Kit, which is not explicitly designed for lipid-rich tissue. This confirmed that the RNeasy Lipid Tissue Mini Kit is better suited to lipid-rich tissue. We then tested the kit on two other genetically different mice that we evaluated on an HFD in a paper under review: Ptenl^−/−^ and Cd68^−/−^ (Table 4). Unexpectedly, we observed lower concentrations in both murine knockout models compared to WT, suggesting that genetic background may influence extraction and thus concentrations. Regardless of the model, we still achieve high purity in total RNA with this kit, making it suitable for RNA sequencing but not for RT-qPCR.

### 4.3. Optimized Protocol Combining TRIzol and Chloroform

First, we tested a protocol combining TRIzol and chloroform to extract RNA from adipose tissue after an HFD, which has the highest lipid content. We collected tissue samples from Ptenl^−/−^ mice, which were the most challenging to extract in previous tests with the two other kits (Table 5). We observed no clear correlation between adipose tissue weight and concentration. Still, using this protocol, we achieved a higher concentration, although the purity (A_260/280_) was lower than 1.72 in the first samples we tested. However, we observed that heating for 10 min at 55–60 °C increased the concentration but did not improve the purity.

**Figure 6:**
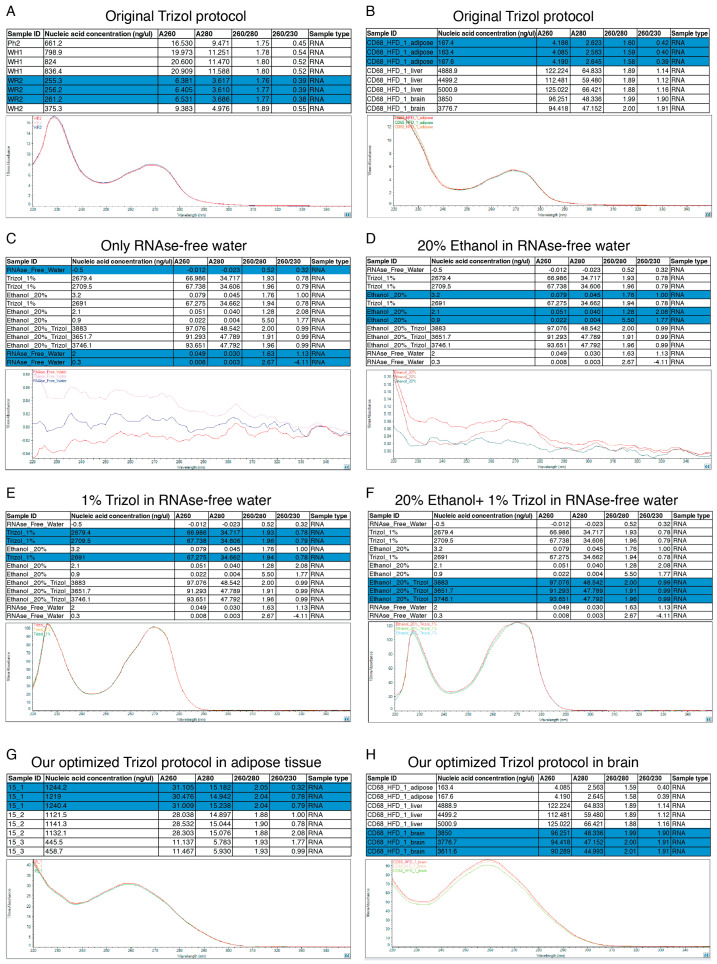
Absorbance ratio curves. (**A**) An example of an extraction using the original TRIzol protocol with low A_260/280_ and A_260/230_ readings, which shows two peaks. (**B**) Another example of an extraction using the original TRIzol protocol with low A_260/280_ and A_260/230_ readings, showing a slot around A_230_ and a peak. (**C**) An example with only RNase-free water. (**D**) An example with 20% ethanol in RNase-free water, showing a slot between A_220_ and A_230_ and a slight peak around A_270_. (**E**) An example with 1% TRIzol in RNase-free water, showing two peaks around A_225_ and A_270_. (**F**) An example with 20% ethanol and 1% TRIzol in RNase-free water, showing two peaks around A_230_ and A_275_. (**G**) An example of an extraction using our optimized TRIzol protocol for adipose tissue with high A_260/280_ and better A_260/230_ readings, which shows a slight slope and a peak around A_260_. (**H**) An example of an extraction using our optimized TRIzol protocol for brain tissue with high A_260/280_ and A_260/230_ readings, which shows the smallest slot and a peak around A_260_. In each panel, the blue highlights indicate the data displayed in the curve below, which represents expected absorbance patterns. There are three replicates per condition.

Because the RNA A_260/280_ purity is below 1.7 and the A_260/230_ ratio is below 1, we cannot use these samples for follow-up experiments such as RNA-seq and RT-qPCR. The TRIzol user manual indicates that partially dissolved RNA samples have an A_230/280_ ratio below 1.6. To identify the possible cause of this low purity and address it, we examined the absorbance ratios for RNA, DNA, and proteins, A_260/280_ and A_260/230_, which are the standard metrics for purity using NanoDrop spectrophotometers. An A_260/280_ below 2 indicates the presence of protein contamination, whereas an A_260/230_ below 2 indicates contamination in salts, phenol, or ethanol. In their manual, they noted that residual reagent contamination from phenol or TRIzol extraction may be indicated by abnormal spectra between 220 and 240 nm and by shifts in the 260–280 nm region. We evaluated it using a NanoDrop and found that our samples exhibited a profile similar to 1% TRIzol, 20% ethanol, or 1% TRIzol and 20% ethanol, with two peaks, indicating potential contamination with ethanol and TRIzol (Figure 6A–F). We repeated the experiments, carefully discarded the supernatants using a micropipettor, and repeated the ethanol wash step twice. We performed a 15 min heating step at 55–60 °C instead of 10 min to evaporate as much ethanol and isopropanol as possible. By performing this three-step wash and extending the evaporation, we observed a significant improvement in purity and, unexpectedly, also in concentration (Table 6) and in the absorbance ratio curves (Figure 6G,H). The new concentration and purity are sufficient for downstream analysis of total RNA, including RNA-seq and RT-qPCR.

### 4.4. Extraction of Total RNA in Liver and Brain Tissues

We compared our optimized TRIzol protocol across three tissues to determine whether the challenge observed in adipose tissue is also present in two other highly lipid-rich tissues, the brain and the liver. We found that liver tissues have higher concentrations and better quality than brain and adipose tissues (Table 7 and Table 8).

**Figure 7:**
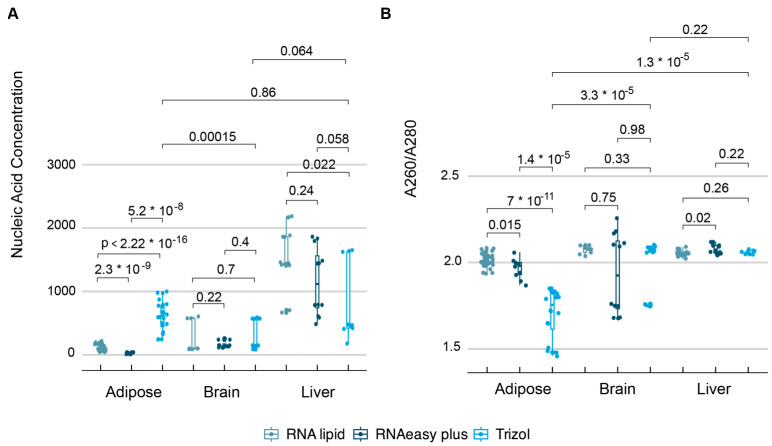
Extraction of total RNA in adipose, liver and brain tissues. (**A**) Nucleic acid concentration in ng/μL. (**B**) Purity of RNA using A_260/280_, starting with an average of 25 mg of tissues. *p*-value taken from a two-sample Wilcoxon test.

To confirm that adipose tissue has the lowest concentration and purity among the three highly lipidic tissues, including the brain and liver, we evaluated these tissues using the three technique replicates in four mice per genetic background (Ptenl^−/−^, and Cd68^−/−^ mice) under an HFD (Figure 7). We validated that liver and brain tissues yielded higher total RNA concentrations and A_260/280_ ratios than adipose tissues. Additionally, we confirmed that the highest concentrations are in the liver, followed by the brain tissue. Regarding the technologies, we found no method that was clearly distinct from the others for liver and brain tissues, confirming that adipose tissue is more complex, with the most highly lipidic tissues affecting the extraction of pure RNA. 

### 4.5. Extraction of Total Proteins in Adipose, Liver, and Brain Tissues

**Figure 8:**
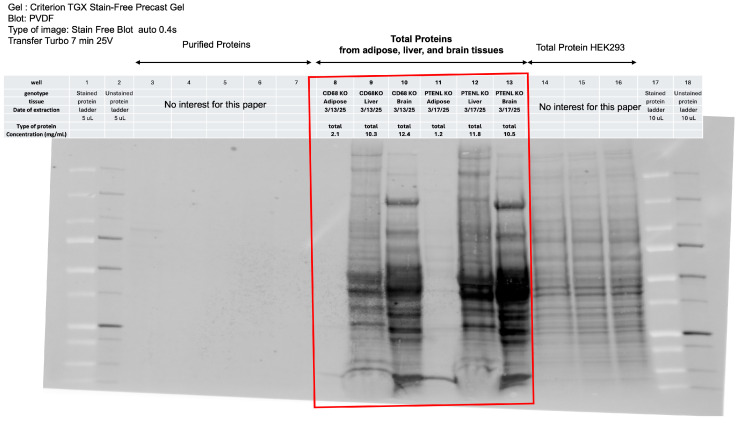
Stain-free imaging during Western blotting that measures total proteins in adipose, liver, and brain tissues. The red square corresponds to a well containing 20 µg of total proteins collected by the Minute Total Protein Extraction Kit for Adipose Tissues/Cultured Adipocytes. Wells 1 and 17 are pre-stained protein ladders, and wells 2 and 18 are unstained protein ladders, which are also activated by UV. The other wells are unrelated to the present protocol: specific purified protein produced in HEK 293FT cells (wells 3–7) and total protein extracted from HEK 293FT after transfection with a plasmid expressing our protein of interest (wells 14–16). We used a stain-free precast gel from Bio-Rad (Hercules, CA, USA), which contains a polyacrylamide gel with a trihalo compound that directly fluorescently labels proteins via short photoactivation, enabling immediate visualization at any point during electrophoresis and Western blotting before transfer to a PVDF membrane for a conventional Western blot.

Because we identified a challenge in RNA extraction from adipose tissue compared with liver and brain tissues, we also evaluated protein extraction from these tissues. First, we thought of testing the RIPA buffer. We observed a limitation with either the Bullet Blender^®^ Homogenizer or the tissue disrupter: they require at least 3 mL of buffer for use, corresponding to 300 mg of tissue. We did not have enough adipose tissue to test it. We therefore test only the Invent Technology kit, which requires less tissue and does not require mechanical tissue disruptors. We obtained high protein concentrations from the tissues; however, we observed that adipose tissue contains fewer total proteins than the brain and liver (Table 9). To observe the distribution of total proteins, we used a stain-free gel, which enables detection of protein bands in gels and on transfer membranes without the use of colorimetric or fluorescent stains [44]. Stain-free imaging during Western blotting, despite adding 20 μg of total protein per well, revealed a different distribution of total proteins, with lower detection in adipose tissues (Figure 8).

**Figure 9:**
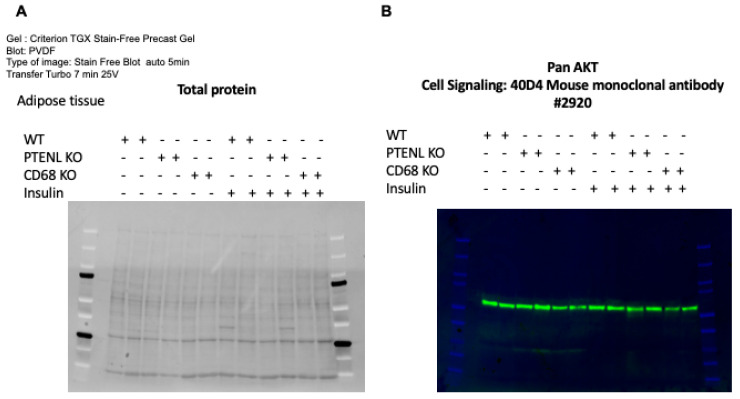
Western blot on adipose tissue. (**A**) Stain-free imaging during Western blotting that measures total proteins in adipose tissues from WT, PTENL^−/−^, and CD68^−/−^ mice with or without insulin IP injection. (**B**) Composite image obtained by ChemiDoc, consisting of chemiluminescence, which measures the panAKT protein level using HRP secondary antibody, and colorimetry, which shows pre-stained protein ladders.

To assess whether we can detect proteins in adipose tissue via Western blotting, we injected insulin intraperitoneally (I.P.) and collected adipose tissue 15 min later. We performed Western blotting using a Criterion TGX stain-free precast gel with 50 μg of total protein per well, transferred the blot to a PVDF membrane, and utilized stain-free imaging before measuring panAKT levels with an antibody and chemiluminescence. When we filled the wells with only total protein from adipose tissues and applied longer UV stimulation to measure total protein, we observed similar total protein compositions across genotypes and stimulation conditions. After incubating the membranes with the primary panAKT antibody, followed by an HRP-conjugated secondary antibody, we detected similar panAKT protein levels in WT and KO mice, with or without insulin stimulation, as expected (Figure 9). This indicates that the total protein extracted from adipose tissue using the commercial kit can be used for Western blotting and for measuring proteins of interest without altering the Western blotting protocol.

## 5. Discussion

A deeper understanding of adipose tissue is essential for advancing treatments for patients with obesity and other diseases worsened by being overweight, such as cancer and cardiovascular diseases. There is growing recognition that studying these diseases should employ a systems biology approach that examines both affected tissues and distant organs that may play significant roles. Although adipose tissue plays a vital role in several diseases, few studies have examined it due to challenges in managing this lipid-rich tissue. In this study, we compared two commercial methods with the optimized protocol we developed for transcript extraction from adipose tissue and a commercial method with the classical protocol using RIPA buffer for protein extraction from adipose tissue. We also assessed their performance in liver and brain tissues, two other primary lipid-rich tissues, to ensure broader applicability.

Although commercial RNA extraction methods yielded high-quality data from brain and liver samples, we encountered additional challenges in obtaining accurate concentrations and purities in adipose tissue for downstream analysis. Multiple factors, such as lipid content, complicate the study of these tissues [23]. Lipids cause clogging of filters, columns, and pipette tips and interfere with enzymatic reactions [25,26,27]. We believe that the low concentrations obtained by commercial transcript extraction methods are due to the use of column tubes with filters that are clogged by high-lipid-content tissues. These challenges have also been recognized and, to some degree, mitigated previously through optimized protocols [7,9,31,33,34,35]. We tested the previously recommended optimized protocols. However, they showed inconsistent or insufficient RNA yield, purity, and/or integrity, which complicates RNA extraction from adipose samples. RNA is more fragile than proteins, making it more susceptible to degradation during extraction, particularly due to factors such as lipids, heat, and RNases [1]. Our difficulty in replicating previously proposed optimized protocols may stem from the fact that those papers were primarily results-focused rather than practical methodological guides, underscoring the need for such protocol publications. In addition, we observed more challenges in extracting transcripts from KO genotypes than from WT mice. Consequently, KO could affect the size and number of lipid droplets in adipose tissue under HFD. Indeed, in adipose tissue of equal weight, larger or more numerous lipid droplets within cells reduce cell numbers, thereby lowering overall RNA and protein yields [45]. We recommend that readers follow our approach: they should first test their practice of our optimized protocol on the liver and brain and then on adipose tissue in WT mice to troubleshoot protocol application and build confidence in applying it to their genotypes.

We optimized the TRIzol protocol, which offers more flexibility than other kits, to minimize RNA degradation and impurities from either frozen tissues stored in tubes with snap-frozen solution, which can be replaced with nitrogen, or fresh tissues. First, we found that previous protocols did not sufficiently emphasize keeping solutions and buffers at low temperature for most steps, while a few steps require room-temperature (21–23 °C) conditions to be active. This may be a simple error that could reduce yield and quality, but it is easy to fix by providing clearer instructions in the protocols [46], as we did here. Additionally, adipose tissue presents unique biochemical and physical barriers to nucleic acid and protein isolation compared with liver, muscle, or brain tissue. These challenges arise from (1) extreme lipid abundance, (2) lipid droplet architecture, (3) low cytoplasmic volume per cell, (4) high endogenous RNase activity, and (5) buoyancy-driven phase partitioning during extraction. Adipocytes are specialized lipid storage cells with minimal cytoplasmic volume [47]. Consequently, adipose tissue contains more lipids per cell and has a lower overall organ weight; RNA and protein concentrations per unit organ weight are lower than in other tissues [23,48]. We improve the original TRIzol protocol to answer these challenges.

First, mature adipocytes contain a single, large unilocular lipid droplet surrounded by a phospholipid monolayer coated with perilipin family proteins [49]. This structure compresses the cytoplasm and nucleus into a thin peripheral rim. Lipid droplets are not inert fat depots; they are protein-coated organelles with regulated interfaces. This architecture creates two problems: mechanical resistance to homogenization and incomplete lysis if disruption is insufficient. Because most of the cell volume consists of neutral lipids, standard homogenization may not efficiently disrupt the thin cytoplasmic and nuclear rims that contain RNA. Moreover, adipose tissue expresses secreted and intracellular RNases, which can compromise RNA integrity during slow or incomplete lysis [6,7,8,9]. Additionally, stress and inflammation, which are common in obese adipose tissue, can increase RNase levels and oxidative stress, thereby further jeopardizing RNA integrity [49]. To address this, our optimized protocol proposes using more aggressive mechanical disruption (bead beating or rotor-stator homogenization) and emphasizes maintaining low tissue temperatures and minimizing processing time. The use of TRIzol reagent, a high-guanidinium solution, is also employed to rapidly denature RNases and collapse protein–RNA interactions.

As previously described, adipose tissue has a higher lipid content and a smaller cytoplasmic volume than most other tissues, and such cells with high lipid content are prone to rupture [28,47]. High neutral lipid content promotes the formation of persistent lipid layers during phenol–chloroform or TRIzol extraction, thereby trapping nucleic acids at the interphase and reducing recovery. Lipids can also contaminate phases, inhibit reverse transcription (e.g., RT-qPCR), and interfere with other enzymatic reactions [26,29,30,31]. To address this challenge, we use higher-speed centrifugation to improve lipid layer separation and propose optional steps to remove the floating fat layer prior to phase collection to reduce organic contamination. To facilitate this improvement and increase yield, it is important to thoroughly mix chloroform and TRIzol to ensure efficient component interactions before performing high-speed centrifugation to separate RNA from DNA and proteins.

We identified another set of critical steps to increase the yield, which involve using precooled isopropanol to maintain the aqueous phase at a low temperature, thereby preventing RNA degradation, and extending the incubation at 4 °C for at least 15 min to ensure sufficient RNA precipitation. By extending the isopropanol incubation, which increases yield, we also increase the risk of salt co-precipitation, making a thorough wash with 75% ethanol crucial. In fact, 75% ethanol effectively removes residual salts (e.g., sodium acetate) and other contaminants from the RNA pellet without dissolving the RNA itself. This works because the 25% water content dissolves inorganic salts, while the high alcohol concentration keeps the RNA in an insoluble precipitate. Using a higher centrifuge speed also facilitates the separation of the RNA-rich insoluble precipitate from the salt-containing aqueous phase. Overall, we achieved higher concentrations than those reported in previous protocols for low-weight tissues (more than 300 ng/µL from less than 100 mg of tissue) and high purity (above 1). Additional ethanol washes could increase yield and purity. Therefore, even if adipose tissue contains fewer cells with a higher risk of RNA degradation and lower overall RNA quality and quantity, our RNA-optimized protocol with TRIzol ensures more complete cell lysis and RNA release while preventing RNA degradation by inhibiting RNase activity and reducing impurities through multiple ethanol wash steps.

Interestingly, for the extraction of proteins from adipose tissues, we found a commercial column-based kit, the Minute Total Protein Extraction Kit for Adipose Tissues/Cultured Adipocytes from Invent Technology, which meets our expectations for allowing us to perform Western Blot and multiplex cytokine assays with simple, quick steps, using less than 100 mg of tissue [50]. This commercial column-based kit does not pose a risk of filter clogging, which could be prevented by the initial step using protein extraction powder. Therefore, we did not suggest any changes to this protocol and used it as is in our research paper to perform Western blot and Luminex assays. We observed lower total protein detection on the stain-free gel in adipose tissue than in the liver and brain, even when the same amount of total protein was applied. However, we encountered no difficulty detecting proteins with antibodies during the subsequent steps of the Western blotting protocol. The trihalo compound, immobilized in a stain-free polyacrylamide gel, is covalently bound to tryptophan residues, enhancing their fluorescence upon UV irradiation and thereby enabling the detection of proteins at levels as low as 10–25 ng. The low detection of total protein by UV light may indicate lower concentrations at specific bands, as observed in the brain and liver, which exhibit distinct protein profiles. This highlights the need for further studies on the protein composition of adipose tissue compared with that of other organs.

## 6. Conclusions

In conclusion, we provide more straightforward, effective, and accessible protocols for extracting total RNA and proteins from adipose, brain, and liver tissues, even on a high-fat diet that increases the number and size of lipid droplets in cells, without requiring extensive sample preparation. Because adipose tissue contains more lipids per cell and has a lower overall organ weight, the concentrations of RNA and protein per unit organ weight are lower than in other tissues, and traditional protocols for extracting total RNA and protein are less sensitive. The optimized protocol using TRIzol and chloroform, along with the Minute Total Protein Extraction Kit for adipose tissues/cultured adipocytes, works on adipose tissues and can be used on less lipid-rich tissues, such as brain and liver, while still providing a higher yield than the classical approach and good purity for downstream analysis. These protocols can be easily adapted to test other highly lipidic tissues (e.g., bone marrow) in mice, humans, and other animals, making them valuable tools in obesity research and other studies involving these tissues.

## Figures and Tables

**Table 1:** Highly lipidic tissues. Lipid content by weight can be higher in specific contexts, such as a high-fat diet, particularly in white adipose tissue and the liver in humans (#) and mice (*).

Tissue	Lipid by Wet Weight
White adipose tissue	60–85% (#*) [10,11]
Yellow bone marrow (mainly adipocytes)	60–80% (#) [12]
Brown adipose tissue	40–60% (#) [11]
Brain white matter	10–15% but (~60% dry weight lipid) (#) [13]
Liver (normal)	5–10% (up to 40% in steatosis) (#*) [14,15,16]
Adrenal/ovary/testis (steroidogenic tissues)	2–10% (#*) [17,18]
Other mixed tissues (skin, mammary gland, muscle, red bone marrow)	5–25% depending on state (#*) [19,20,21,22]

**Table 2:** Extraction of total RNA in adipose from WT mouse on an HFD. Standard deviation (St. Dev.) calculated from three technical replicates.

Sample	S1	S2	S3
Concentration (ng/μL)	11.37	33.7	41.7
St. Dev. concentration	0.9	1.5	0.6
Purity (A260/A280)	1.97	1.92	1.99
St. Dev. purity	0.1	0.03	0.02
Total RNA (ng)	568.5	1685	2085
Final yield (ng of RNA/mg of tissue)	56.85	56.16	20.85
Volume used to elute (μL)	50	50	50
Weight tissue used (mg)	10	30	100

**Table 3:** Extraction of total RNA in adipose from WT mice on an HFD and RD. Standard deviation (St. Dev.) calculated from three technical replicates.

Sample	S4	S5	S6	S7
Diet	HFD	RD	HFD	HFD
Concentration (ng/μL)	105.3	96	204.4	183.2
St. Dev. concentration	0.7	1.04	10.6	1.5
Purity (A260/A280)	2	2.03	2.07	2.06
St. Dev. purity	0.005	0.005	0.006	0.01
Total RNA (ng)	3165	2865	6132	5496
Final yield (ng of RNA/mg of tissue)	63.3	57.3	102.2	54.96
Volume used to elute (μL)	30	30	30	30
Weight tissue used (mg)	50	50	60	100

**Table 4:** Extraction of total RNA in adipose from Ptenl^−/−^ and Cd68^−/−^ mice on an HFD and RD. Standard deviation (St. Dev.) calculated from three technical replicates.

Sample	S8	S9	S10	S11	S12	S13	S14
Genetic background	Cd68^−/−^	Cd68^−/−^	Cd68^−/−^	Cd68^−/−^	Ptenl^−/−^	Ptenl^−/−^	Ptenl^−/−^
Diet	RD	HFD	HFD	HFD	HFD	HFD	RD
Concentration (ng/μL)	61.8	176.5	139.5	73.3	44.3	60.7	59.2
St. Dev. concentration	0.85	11.7	0.7	1.8	1.4	2	1.9
Purity (A260/A280)	2.03	2	2	2	1.99	1.95	1.97
St. Dev. purity	0.03	0.04	0.07	0.01	0.005	0.02	0.03
Total RNA (ng)	1881	5295	4185	2100	1329	1821	1806
Final yield (ng of RNA/mg of tissue)	37.62	105.9	83.7	35	26.58	36.42	25.8
Volume used to elute (μL)	30	30	30	30	30	30	30
Weight tissue used (mg)	50	50	50	60	50	50	70

**Table 5:** Extraction of total RNA in adipose from Ptenl^−/−^ mice on an HFD using 2 mL of TRIzol. Standard deviation (St. Dev.) calculated from three technical replicates.

Sample	S15	S16	S15	S16
Heating (55–60 °C)	No	No	Yes	Yes
Concentration (ng/μL)	104.3	191.2	244.6	341.03
St. Dev. concentration	11.4	2.66	1.82	16.75
Purity (A260/A280)	1.66	1.68	1.65	1.7
St. Dev. purity	0.01	0.005	0	0.005
Purity (A260/A230)	0.55	0.60	0.89	0.65
Total RNA (ng)	2086	3824	4892	6820.6
Final yield (ng of RNA/mg of tissue)	24.54	41.56	57.55	74.13
Volume used to elute (μL)	20	20	20	20
Weight tissue used (mg)	85	92	85	92

**Table 6:** Extraction of total RNA in adipose from WT, Ptenl^−/−^, and Cd68^−/−^ mice under RD and HFD. Standard deviation (St. Dev.) calculated from three technical replicates.

Sample	S17	S18	S19	S20	S21	S22
Genetic background	Ptenl^−/−^	Ptenl^−/−^	WT	WT	Cd68^−/−^	Cd68^−/−^
diet	HFD	RD	HFD	RD	HFD	RD
Concentration (ng/μL)	659.03	486.6	816.8	775.9	588.73	984.4
St. Dev. concentration	21.3	14.4	48.6	14.8	9.57	13.7
Purity (A260/A280)	1.49	1.83	1.84	1.83	1.49	1.79
St. Dev. purity	0.01	0	0.01	0.01	0.01	0.05
Purity (A260/A230)	0.38	0.76	0.91	0.55	0.34	0.72
Total RNA (ng)	13,180.6	9732	16,336	15,518	3530	19,688
Final yield (ng of RNA/mg of tissue)	151.5	109	209.4	189.3	44.7	207.3
Volume used to elute (μL)	20	20	20	20	20	20
Weight tissue used (mg)	87	89	78	82	79	95

**Table 7:** Extraction of total RNA in adipose, liver, and brain tissue from the same Ptenl^−/−^ mouse on an HFD using our optimized protocol with TRIzol and chloroform. Standard deviation (St. Dev.) calculated from three technical replicates.

Tissue	Adipose	Liver	Brain
Concentration (ng/μL)	386.5	4099.5	1532.3
St. Dev. concentration	11.02	76.44	38.35
Purity (A260/A280)	1.55	1.94	1.96
St. Dev. purity	0.03	0.01	0.01
Purity (A260/A230)	0.63	1.40	1.49
Total RNA (ng)	7730	81,990	30,646
Final yield (ng of RNA/mg of tissue)	82.23	1037.8	348.25
Volume used to elute (μL)	20	20	20
Weight tissue used (mg)	94	79	88

**Table 8:** Extraction of total RNA in adipose, liver, and brain tissue from the same Cd68^−/−^ mouse on an HFD using our optimized protocol with TRIzol and chloroform. Standard deviation (St. Dev.) calculated from three technical replicates.

Tissue	Adipose	Liver	Brain
Concentration (ng/μL)	166.1	4796.3	3746.1
St. Dev. concentration	2.4	263.3	1221
Purity (A260/A280)	1.59	1.88	2
St. Dev. purity	0.01	0.005	0.01
Purity (A260/A230)	0.40	1.14	1.9
Total RNA (ng)	3322	95,926	74,922
Volume used to elute (μL)	20	20	20

**Table 9:** Extraction of total protein in adipose, liver, and brain from Ptenl^−/−^ and Cd68^−/−^ mice under HFD. Measurement of concentration using the BCA assay.

Sample	S23	S23	S23	S24	S24	S24
Genetic background	*Ptenl^−/−^*	*Ptenl^−/−^*	*Ptenl^−/−^*	*Cd68^−/−^*	*Cd68^−/−^*	*Cd68^−/−^*
diet	HFD	HFD	HFD	HFD	HFD	HFD
tissue	adipose	liver	brain	adipose	liver	brain
Concentration (mg/mL)	2.1	12.4	10.3	1.2	11.8	10.5
Total protein (ng)	210	1240	1030	120	1180	1050
Final yield (ng of protein/mg of tissue)	2.25	20.6	14.7	1.66	14.6	13.5
Volume used to elute (μL)	100	100	100	100	100	100
Weight tissue used (mg)	93	60	70	72	81	78

## Data Availability

The original contributions presented in this study are included in the article. Further inquiries can be directed to the corresponding author.

## Abbreviations

The following abbreviations are used in this manuscript:

HFD	High-fat diet
RD	Regular diet

## References

[1] Aronne L.J., Horn D.B., le Roux C.W., Ho W., Falcon B.L., Valderas E.G., Das S., Lee C.J., Glass L.C., Senyucel C. (2025). Tirzepatide as Compared with Semaglutide for the Treatment of Obesity. N. Engl. J. Med..

[2] Lincoff A.M., Brown-Frandsen K., Colhoun H.M., Deanfield J., Emerson S.S., Esbjerg S., Hardt-Lindberg S., Hovingh G.K., Kahn S.E., Kushner R.F. (2023). Semaglutide and Cardiovascular Outcomes in Obesity without Diabetes. N. Engl. J. Med..

[3] Neeland I.J., Linge J., Birkenfeld A.L. (2024). Changes in Lean Body Mass with Glucagon-Like Peptide-1-Based Therapies and Mitigation Strategies. Diabetes Obes. Metab..

[4] Applegate C.C., Kang Y., Deng H., Chen D., Medina N.Y.G., Cui Y., Feng Y., Kuo C.-W., Shahoei S.H., Kim H. (2025). Nanomedicine Targeting Ppar in Adipose Tissue Macrophages Improves Lipid Metabolism and Obesity-Induced Metabolic Dysfunction. Sci. Adv..

[5] Hinte L., Castellano-Castillo D., Ghosh A., Melrose K., Gasser E., Noe F., Massier L., Dong H., Sun W., Hoffmann A. (2024). Adipose Tissue Retains an Epigenetic Memory of Obesity after Weight Loss. Nature.

[6] Sakers A., De Siqueira M.K., Seale P., Villanueva C.J. (2022). Adipose-Tissue Plasticity in Health and Disease. Cell.

[7] Zwick R.K., Guerrero-Juarez C.F., Horsley V., Plikus M.V. (2018). Anatomical, Physiological, and Functional Diversity of Adipose Tissue. Cell Metab..

[8] Kwok K.H., Lam K.S., Xu A. (2016). Heterogeneity of White Adipose Tissue: Molecular Basis and Clinical Implications. Exp. Mol. Med..

[9] Chun K.H. (2021). Mouse Model of the Adipose Organ: The Heterogeneous Anatomical Characteristics. Arch. Pharm. Res..

[10] Jeanrenaud B. (1965). Lipid Components of Adipose Tissue. Compr. Physiol..

[11] Cannon B., Nedergaard J. (2004). Brown Adipose Tissue: Function and Physiological Significance. Physiol. Rev..

[12] Vogler J.B., Murphy W.A. (1988). Bone Marrow Imaging. Radiology.

[13] O’Brien J.S., Sampson E.L. (1965). Lipid Composition of the Normal Human Brain: Gray Matter, White Matter, and Myelin. J. Lipid Res..

[14] Folch J., Lees M., Sloane Stanley G.H. (1957). A Simple Method for the Isolation and Purification of Total Lipides from Animal Tissues. J. Biol. Chem..

[15] Browning J.D., Szczepaniak L.S., Dobbins R., Nuremberg P., Horton J.D., Cohen J.C., Grundy S.M., Hobbs H.H. (2004). Prevalence of Hepatic Steatosis in an Urban Population in the United States: Impact of Ethnicity. Hepatology.

[16] Younossi Z.M., Koenig A.B., Abdelatif D., Fazel Y., Henry L., Wymer M. (2016). Global Epidemiology of Nonalcoholic Fatty Liver Disease-Meta-Analytic Assessment of Prevalence, Incidence, and Outcomes. Hepatology.

[17] Sheriff D.S., Govindarajulu P. (1978). The Lipid Composition of Human Testis in Testicular Feminization Syndrome. Horm. Res..

[18] Andersen J.M., Dietschy J.M. (1978). Relative Importance of High and Low Density Lipoproteins in the Regulation of Cholesterol Synthesis in the Adrenal Gland, Ovary, and Testis of the Rat. J. Biol. Chem..

[19] Aoki T., Yamaguchi S., Kinoshita S., Hayashida Y., Korogi Y. (2016). Quantification of Bone Marrow Fat Content Using Iterative Decomposition of Water and Fat with Echo Asymmetry and Least-Squares Estimation (Ideal): Reproducibility, Site Variation and Correlation with Age and Menopause. Br. J. Radiol..

[20] Wertz P.W., Madison K.C., Downing D.T. (1989). Covalently Bound Lipids of Human Stratum Corneum. J. Investig. Dermatol..

[21] Rudolph M.C., McManaman J.L., Phang T., Russell T., Kominsky D.J., Serkova N.J., Stein T., Anderson S.M., Neville M.C. (2007). Metabolic Regulation in the Lactating Mammary Gland: A Lipid Synthesizing Machine. Physiol. Genom..

[22] Goodpaster B.H., He J., Watkins S., Kelley D.E. (2001). Skeletal Muscle Lipid Content and Insulin Resistance: Evidence for a Paradox in Endurance-Trained Athletes. J. Clin. Endocrinol. Metab..

[23] Stroh A.M., Lynch C.E., Lester B.E., Minchev K., Chambers T.L., Montenegro C.F., Martinez C.C., Fountain W.A., Trappe T.A., Trappe S.W. (2021). Human Adipose and Skeletal Muscle Tissue DNA, Rna, and Protein Content. J. Appl. Physiol. (1985).

[24] Liu J., Ni L., Peng M., Liang Y., Lu C., Zheng H., Huang Z., Zhang J., Chen R. (2024). Optical Imaging for Brown or Beige Adipose Tissue. View.

[25] Schrader C., Schielke A., Ellerbroek L., Johne R. (2012). Pcr Inhibitors—Occurrence, Properties and Removal. J. Appl. Microbiol..

[26] Zafeiriadou A., Nano K., Thomaidis N.S., Markou A. (2024). Evaluation of Pcr-Enhancing Approaches to Reduce Inhibition in Wastewater Samples and Enhance Viral Load Measurements. Sci. Total Environ..

[27] Hagberg C.E., Li Q., Kutschke M., Bhowmick D., Kiss E., Shabalina I.G., Harms M.J., Shilkova O., Kozina V., Nedergaard J. (2018). Flow Cytometry of Mouse and Human Adipocytes for the Analysis of Browning and Cellular Heterogeneity. Cell Rep..

[28] Di Rocco G., Trivisonno A., Trivisonno G., Toietta G. (2024). Dissecting Human Adipose Tissue Heterogeneity Using Single-Cell Omics Technologies. Stem Cell Res. Ther..

[29] Cirera S. (2013). Highly Efficient Method for Isolation of Total Rna from Adipose Tissue. BMC Res. Notes.

[30] Nandi Jui B., Sarsenbayeva A., Jernow H., Hetty S., Pereira M.J. (2022). Evaluation of Rna Isolation Methods in Human Adipose Tissue. Lab. Med..

[31] Zhang H., Liu Y., Yu B., Lu R. (2023). An Optimized Trizol-Based Method for Isolating Rna from Adipose Tissue. Biotechniques.

[32] Borgeson E., Boucher J., Hagberg C.E. (2022). Of Mice and Men: Pinpointing Species Differences in Adipose Tissue Biology. Front. Cell Dev. Biol..

[33] Song L., Lee C., Schindler C. (2011). Deletion of the Murine Scavenger Receptor Cd68. J. Lipid Res..

[34] Riquelme S.A., Hopkins B.D., Wolfe A.L., DiMango E., Kitur K., Parsons R., Prince A. (2017). Cystic Fibrosis Transmembrane Conductance Regulator Attaches Tumor Suppressor Pten to the Membrane and Promotes Anti Pseudomonas Aeruginosa Immunity. Immunity.

[35] Bagchi D.P., MacDougald O.A. (2019). Identification and Dissection of Diverse Mouse Adipose Depots. J. Vis. Exp..

[36] Galmozzi A., Kok B.P., Saez E. (2021). Isolation and Differentiation of Primary White and Brown Preadipocytes from Newborn Mice. J. Vis. Exp..

[37] Mortimer T., Welz P.S., Benitah S.A., Koronowski K.B. (2021). Collecting Mouse Livers for Transcriptome Analysis of Daily Rhythms. STAR Protoc..

[38] Collins S.C., Wagner C., Gagliardi L., Kretz P.F., Fischer M.C., Kessler P., Kannan M., Yalcin B. (2018). A Method for Parasagittal Sectioning for Neuroanatomical Quantification of Brain Structures in the Adult Mouse. Curr. Protoc. Mouse Biol..

[39] MacManus D.B. (2023). Excision of Whole Intact Mouse Brain. MethodsX.

[40] Beaudoin G.M., Lee S.H., Singh D., Yuan Y., Ng Y.G., Reichardt L.F., Arikkath J. (2012). Culturing Pyramidal Neurons from the Early Postnatal Mouse Hippocampus and Cortex. Nat. Protoc..

[41] Qiagen (2019). Rneasy^®^ Lipid Tissue Mini Handbook.

[42] Qiagen (2023). Rneasy^®^ Mini Handbook.

[43] Aranda P.S., LaJoie D.M., Jorcyk C.L. (2012). Bleach Gel: A Simple Agarose Gel for Analyzing Rna Quality. Electrophoresis.

[44] Biorad (2025). The Complete Guide to Stain-Free Western Blotting.

[45] Maniyadath B., Zhang Q., Gupta R.K., Mandrup S. (2023). Adipose Tissue at Single-Cell Resolution. Cell Metab..

[46] Teixeira da Silva J.A. (2021). Room Temperature in Scientific Protocols and Experiments Should Be Defined: A Reproducibility Issue. Biotechniques.

[47] Cinti S. (2005). The Adipose Organ. Prostaglandins Leukot. Essent. Fatty Acids.

[48] Janke J., Engeli S., Gorzelniak K., Sharma A.M. (2001). Extraction of Total Rna from Adipocytes. Horm. Metab. Res..

[49] Walther T.C., Farese R.V. (2012). Lipid Droplets and Cellular Lipid Metabolism. Annu. Rev. Biochem..

[50] Invent Biotechnologies Inc. (2017). High Efficiency Protein Extraction from Adipose Tissues. Biotechniques.

